# Elevational Gradient in Species Richness Pattern of Epigaeic Beetles and Underlying Mechanisms at East Slope of Balang Mountain in Southwestern China

**DOI:** 10.1371/journal.pone.0069177

**Published:** 2013-07-18

**Authors:** Xiao-Dong Yu, Liang Lü, Tian-Hong Luo, Hong-Zhang Zhou

**Affiliations:** Key Laboratory of Zoological Systematics and Evolution, Institute of Zoology, Chinese Academy of Sciences, Beijing, P. R. China; University of Guelph, Canada

## Abstract

We report on the species richness patterns of epigaeic beetles (Coleoptera: Carabidae and Staphylinidae) along a subtropical elevational gradient of Balang Mountain, southwestern China. We tested the roles of environmental factors (e.g. temperature, area and litter cover) and direct biotic interactions (e.g. foods and antagonists) that shape elevational diversity gradients. Beetles were sampled at 19 sites using pitfall traps along the studied elevational gradient ranging from 1500 m–4000 m during the 2004 growing season. A total of 74416 specimens representing 260 species were recorded. Species richness of epigaeic beetles and two families showed unimodal patterns along the elevational gradient, peaking at mid-elevations (*c.* 2535 m), and the ranges of most beetle species were narrow along the gradient. The potential correlates of both species richness and environmental variables were examined using linear and second order polynomial regressions. The results showed that temperature, area and litter cover had strong explanatory power of beetle species richness for nearly all richness patterns, of beetles as a whole and of Carabidae and Staphylinidae, but the density of antagonists was associated with species richness of Carabidae only. Multiple regression analyses suggested that the three environmental factors combined contributed most to richness patterns for most taxa. The results suggest that environmental factors associated with temperature, area and habitat heterogeneity could account for most variation in richness pattern of epigaeic beetles. Additionally, the mid-elevation peaks and the small range size of most species indicate that conservation efforts should give attention to the entire gradient rather than just mid-elevations.

## Introduction

Variation of species richness patterns along environmental gradients has long been of interest to biologists, and understanding the mechanisms underlying that variation is one of the fundamental questions in ecology [1], [2]. Of these patterns, species richness along the latitudinal gradient is the most striking and perhaps best documented pattern [3], [4], and many hypotheses and underling mechanisms have been proposed to explain it, such as energy availability, evolutionary time, habitat heterogeneity, area and geometric constraints [3], [5], [6].

Elevational gradients in species richness, which are often considered as a mirror of latitudinal pattern at the smaller scale, perhaps offer many characteristics that make them more suitable for uncovering the mechanisms that shape patterns of biodiversity [7], [8]. However, contrasted with monotonic decrease of species richness along the elevation-richness relationship, the elevational pattern of species richness with complexity depends on the particular taxonomical group, and the scale and extent of the elevational gradients [2], [7], [9], [10].

Ecologists and biogeographers have examined gradients of species richness and observed four contrasting patterns: (1) a continuous decline in species richness with elevation, (2) a mid-elevation peak in species richness, (3) no change in species richness, and (4) an increase in species richness with elevation [9], [10]. A unimodal (mid-elevation peak) pattern and monotonic declines in richness with increasing elevation are the two most commonly observed patterns of species richness [9], [10]. Various hypotheses (e.g. the productivity hypothesis, the harsh environment hypothesis, the species-area hypothesis and the resources diversity hypothesis etc.) have been proposed to explain these elevational pattern of species richness [8], [9], [10]. Of these, the most frequently documented correlates and drivers of elevational patterns of diversity are contemporary climates including temperature and precipitation (e.g. water-energy dynamics, MTE) [11], [12], spatial factors including geometric constraints (e.g. the mid-domain effect, MDE) [13] and area size [14], biological processes such as habitat heterogeneity, productivity and interspecific interactions [1], [15], and evolutionary and historical processes such as niche conservatism, isolation, speciation, endemism, and evolutionary diversification [2], [16], [17], [18]. However, most hypotheses or knowledge on species richness and diversity along elevational gradients are based on plants and vertebrates (particularly for mammals and birds) [9], [10], [11]. As the global majority of terrestrial organisms, just a few studies on insects have been examined richness patterns along elevational gradients, focusing on specific taxa such as ants [18], [19], butterflies [20], moths [21], [22], and dung beetles [23], [24], and knowledge of elevational richness patterns and underlying causes for most insects are still very poor.

Epigaeic (or ground-dwelling) beetles (Coleoptera: Carabidae and Staphylinidae) are abundant and diverse, easily sampled, relatively well-known taxonomically and sensitive to habitat changes, and have been frequently adopted as ecological or biodiversity indicators [25], [26]. In addition, carabid and staphylinid beetles are widely distributed while showing association with specific habitat types, thus the use of more than one taxon has the advantage of testing the generality of any observed patterns. But until now, although epigaeic beetles have been extensively used as ecological indicators to help evaluate conservation of biodiversity in landscapes subjected to forest management, habitat fragmentations and human disturbance [27], few studies on these beetles that span over the entire elevational gradient have been conducted to examine elevational richness pattern and underlying causes of the pattern.

In this study, we aim to document, describe, and explain the elevational gradient in epigaeic beetle richness pattern at the east slope of Balang Mountain in southwestern China, belonging to eastern part of Qinghai-Tibet plateau (an established hotspot of biodiversity). Firstly, we describe the pattern along this extensive elevational gradient, and assessed the range size distribution pattern of epigaeic beetles along the elevation gradient by examining the range size of each beetle species. Next, two alternative groups of explanatory factors, environmental (e.g. climate, area, energy, productivity, heterogeneity) and direct biotic interactions (e.g. foods, antagonists), were evaluated to explain the elevational patterns of epigaeic beetle richness. A suite of factors for which data are available and are potential correlates with beetle richness were included in the analysis. These included temperature, precipitation, potential evapotranspiration (PET), actual evapotranspiration (AET), woody species richness, canopy cover, litter cover, and abundances of potential foods (insect larvae) and antagonists (ants). In addition, since the MDE does not provide biological explanations and might bring about a spurious and apparently strong explanatory power for elevational richness patterns [28], we do not include the MDE model into the environment factors.

## Materials and Methods

### Study Area

The study area was located on the east slope of Balang Mountain (also called Balangla in the Tibetan language) of Qionglai Mountains, central Sichuan Province, Southwestern China, a transitional zone between the Sichuan basin and the Tibet plateau. The elevational gradient ranged from the bank of Minjiang River at *c.* 900 m to the summit of Mt. Balang at *c.* 5040 m. Most of the east slope of Mt. Balang was situated on Wolong Natural Reserve (30°45′–31°25′ N and 102°52′–103°24′ E; elevation: 1150–6250 m), with an area of *c.* 2000 km^2^. Since the reserve was created as a base for preserving the Giant Panda in 1975, logging has long been forbidden and the primary (or old-growth) forests and secondary forests are preserved well within the reserve [29]. However, since there are some residents settled in the reserve, the lowlands are partly exploited for agriculture and human disturbance at elevations less than 2200 m. The elevational gradient in the reserve covered six major vegetation zones [29]: evergreen broad-leaved forest (1150–1600 m); evergreen and deciduous broad-leaved mixed forest (1600–2000 m); coniferous and deciduous broad-leaved mixed forest (2000-2700 m); dark coniferous forest (2800–3600 m); alpine shrubs and meadows (3600–4200 m); alpine discontinuous rock and scree vegetation (4200–5000 m).

**Figure 1:**
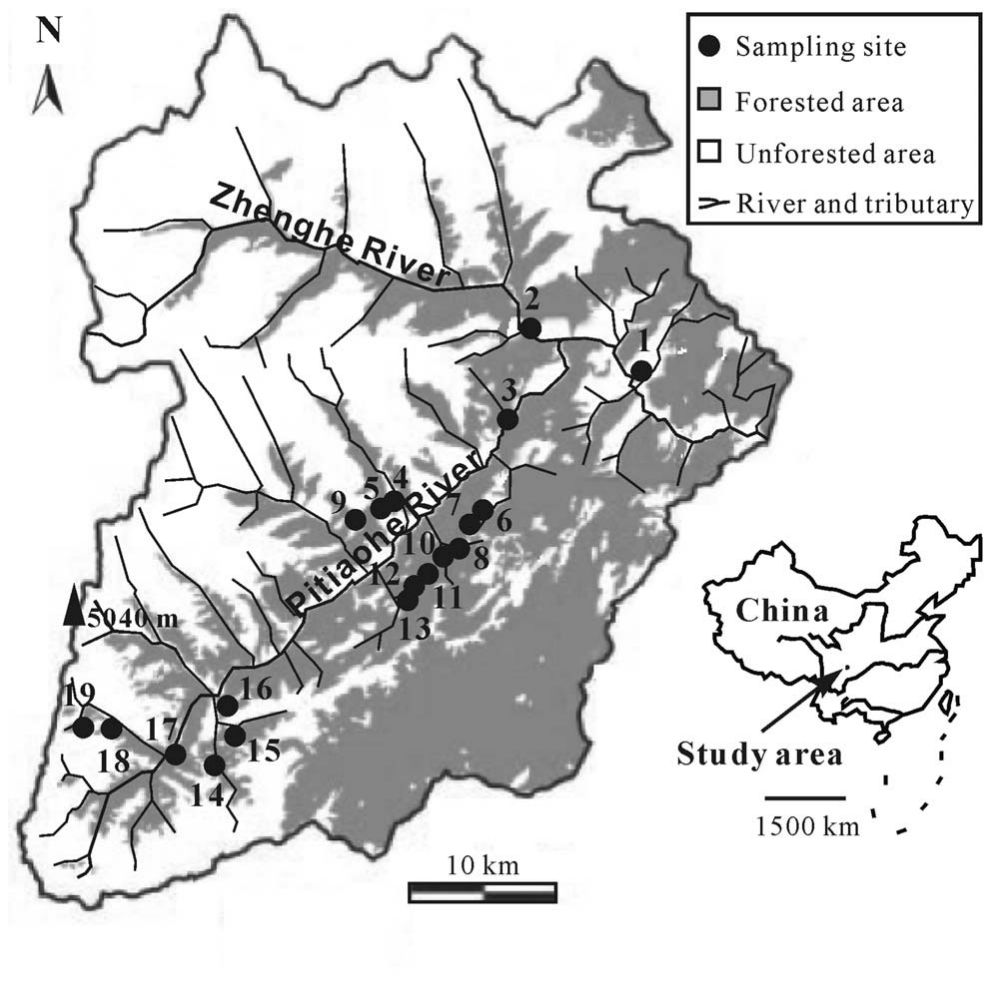
Map of Wolong Natural Reserve showing sampling locations in different elevations. The labels of 1 to 19 represented the sampling sites of 1535 m, 1660 m, 1850 m, 2150 m, 2250 m, 2375 m, 2445 m, 2535 m, 2615 m, 2715 m, 2840 m, 2955 m, 3050 m, 3260 m, 3450 m, 3570 m, 3685 m, 3830 m, and 3950 m a.s.l. along the elevational gradients at east slope of Mt. Balang.

Because the elevational gradients lower than 1400 m and higher than 4200 m are dominated the field or rocks and scree vegetation, respectively, which are not appropriate for the survival of forest species, our study transect on the east slope of Mt. Balang (mainly at Pitiaohe Valley) was set within 1400 m and 4200 m and covered most vegetation types (Figure 1). Fifteen sampling sites were situated at elevations in the well-protected area between 2200 and 4000 m, including 9 sites within natural primeval forests and old secondary forests (2200–3100 m) and 6 sites within alpine shrubs and meadows (3200–4000 m). The alpine meadows were slightly disturbed by tourism. In addition, four sites were placed in the lowlands (agriculture: 1500–2200 m) as the control of higher elevations, which were covered with sparse secondary broad-leaved shrubs, recently planted coniferous trees and dense weeds. So, beetle assemblages were sampled at 19 sites along the elevational gradient, and all the sites were covered with well-protected primeval or old secondary vegetation and located in areas away from roads, heavily visited trails or other recent human disturbances, except the 4 sites in low elevation.

### Beetle Sampling

Considering the disturbance at the elevation higher than 3200 m by tourism and at the elevation lower than 2200 m by agriculture, a strategy of unequal sampling effort was applied at the different studied elevational bands, i.e. only 1 or 2 samples was set at each elevational band in the disturbed sites, and more samples at each elevational band at well-protected and easy-to-reach sites. At each sample, we randomly set a 4-ha plot, and within the plot, four or five subplots (locations) were placed in each plot. In total, there were 58 samples (plots) and 263 subplots in this study (Table S1). For the independence of beetle samples, their distance between plots was 500 m or more, and subplots were 25 m apart from each other [30]. All subplots were set at 100 m from the edge of the plot in order to avoid edge effects.

Beetles were sampled by pitfall traps. Although pitfall traps are biased toward active forms and inaccurate in estimating the absolute density, this method is useful in the monitoring and assessment of local population changes [31]. Each subplot (trapping location) was composed of five traps, which were placed into a cross at a distance of ca. 1 m between traps. Thus, a total of 1315 traps were used in this study (Table S1). Traps were constructed from 400 mL plastic beverage cups (9 cm high by 7.5 diameter). A small hole with the diameter of less than 0.2 cm was drilled on each trap about 2.5 cm below the upper brim, so that excess rainwater could flow out. Each trap was filled with about 100 mL (about 2.5 cm high) of a mixed trapping fluid (vinegar:sugar:alcohol:water, 10 ml:5 g:5 ml:20 ml) to collect beetles. The trapped specimens were preserved in 70% alcohol. Trapping period covered most of the growing season (from the end of April to the beginning of October) in 2004. Traps were emptied and serviced twice a month.

All specimens were deposited in the Insect Museum, Institute of Zoology, CAS. Carabids were identified by Drs. Hong-Bin Liang and Hong-Liang Shi and by comparing specimens against collections at the Institute of Zoology, CAS, and staphylinids by the members of our group through the comparison with original type specimens or other reliably identified material in several museums (see Acknowledgements). The nomenclature follows Lindroth (1961–1969) for Carabidae [32], and Herman (2001) for Staphylinidae [33]. Staphylinids of the subfamily Aleocharinae were excluded from analyses because reliable taxonomic keys or catalogues are unavailable. A full species list is provided in Table S2.

As discussed above, sampling effort (number of samples at each elevation site) and sample size (number of individual beetles collected) varied considerably among sites and samples. Moreover, species accumulation curves approached a plateau for most sites, but richness did not completely saturate for several of them (Figure S1). Therefore, total species richness by counting observed species, which usually depends on total abundance and the number of individuals collected, might be biased as a measure of estimating local species richness [34]. To cope with this problem, three approaches were considered in this study, (1) sample-based rarefied richness, (3) Chao 2 estimated richness, and (3) interpolated (observed) richness. Sample-based rarefied richness could reduce the number of species to below the observed richness for sites with more samples [34]. In this study, we have a minimum of 5 subplots composed of 25 traps per site, so we could rarefy back to 25 traps for each site. The Chao 2 estimate of species richness for the site assuming sampling went to completion are two non-parametric statistical estimators of true local species richness to reduce the bias of incomplete sampling [35], [36]. Interpolated richness with the assumption that each species’ range is continuous along the transect was based directly on the recorded species × site incidence data to fill gaps between lower and upper recorded range limits, whether or not it was actually recorded at intermediate sites [37]. Rarefied richness and Chao 2 estimates were calculated using EstimateS 7.50 [38], and interpolated richness was computed from interpolated species ranges using RangeModel 5 [39].

### Explanatory Variables

To test the role of two contrasting factors (environment vs. direct biotic interactions) on species richness pattern of epigaeic beetles along an elevational gradient, a suite of variables for which data are available and might be correlated with beetle richness were examined in this study. Environment factors included climate and climatic variables (temperature, precipitation, potential evapotranspiration [PET], actual evapotranspiration [AET]), habitat heterogeneity variables (e.g. woody plant species, canopy cover and litter cover), and spatial variables (area). Abundances of potential foods (insect larvae) and antagonists (ants) were used as direct biotic interactions.

#### Climate and climatic variables

Annual temperature and precipitation data used in this study were extracted from earlier climate studies and synthesized based on three years of climate records (2003–2005) from the meteorological station of Sichuan Academy of Forestry at Desheng (*c.* 2745 m) located on Wolong Natural Reserve [40], [41]. According to early published data, temperature decreased at an empirical lapse rate of 0.44°C/100 m, but precipitation increased at a rate of 20.16 mm/100 m with increasing elevation at Balang Mountain [29]. So without the true temperature and precipitation records for the studied sites, annual temperature and precipitation were linearly estimated based the data from the station and the empirical lapse rate along the elevational gradient mentioned above (Figure S2a, d).

In addition, we also calculated potential evapotranspiration (PET) and actual evapotranspiration (AET) as explanatory variables (Figure S2b, c). PET is defined as the amount of evaporation that would occur if a sufficient water source were available, and can be thought as a surrogate of energy. PET can be calculated using the Thornthwaite water balance method using the following formula [42]: *E* = 1.6(10*T*/*I*)*^α^*, where *E = * monthly potential evapotranspiration (cm), *T = * mean monthly temperature, *I = * a heat index for a given area which is the sum of 12 monthly index values *I*, *i* is derived from mean monthly temperatures using the following formula: *i* =  (*T*/5)^1.514^, *α* = an empirically derived exponent which is a function of *I*, *α* = (6.75*10^−7^)*I* ^3^–7.71*10^−5^)*I* ^2^+(1.79* 10^−2^)*I*+0.49. AET is the quantity of water that is actually removed from a surface due to the processes of evaporation and transpiration, and can be considered as surrogate of productivity. We calculated AET using the Turc’s formula, where AET = *P*/[0.9+(*P*/*L*) ^2^]^1/2^, with *L* = 300+25T+0.05T^3^, where *P* = mean annual precipitation, and T = mean annual temperature [43], [44].

#### Habitat heterogeneity variables

The woody plant richness data were extracted from the early investigation during the years of 1979–1982 organized by Wolong Natural Reserve [29]. All woody plant species plots were set as the same or near locations with our beetle sampling, and the missed data of woody species richness in the lowland sites and two highest elevations were interpolated by fitting polynomial functions to their data over the complete transect. Overall woody species richness peaks at a middle elevation between 2500 to 2600 m (*c.* 2535 m) in coniferous and deciduous broad-leaved mixed forest and decreases towards both ends of the gradient (Figure S1f). However, although the establishment of the reserve in 1975 prohibited logging and heavy disturbance from agriculture and improved the quality of vegetation, the woody plant species data on vegetation based on the investigation in 1979–82 might have changed drastically by the time beetle sampling were carried out in 2004. Hence, correlation of data with large scale time difference (around 25 years) might not give correct conclusions, and should be examined carefully.

The percentage data for the canopy cover and litter cover were measured by visual estimation within a radius of 2 m around the center of each trapping location (Figure S2g, h).

#### Spatial variable (area)

The relationship between area and elevation was determined by calculating the number of square kilometres in an elevational band. The study zone in east slope of Mt. Balang was circumscribed within a mostly enclosed valley (Pitiaohe Valley) and its surrounding slopes. Since if the surrounding boundary (ridge) is too low, it can not prevent beetles from moving to the next valley, and we included the next valley(s) at lower elevations and set the nearest ridge more than 4000 m as an effective boundary. To calculate the area at each elevational band in the study zone, we extracted the Digital Elevation Model (DEM) data of ca. 1066 km^2^ of the study zone, with a grid spacing of 8.331312×10^−4^ arc-degree, from the GeoTiff file using the software Global Mapper v12 for trial, and then estimated both the spherical surface areas and the landscape surface areas along the ascending elevational gradients from 1000 m to 4800 m at interval of 100 m. The landscape surface areas were computed directly from DEM with the method described by Jenness (2004) [45]. The GeoTiff file covering the study plots was downloaded from Shuttle Radar Topography Mission (SRTM) (http://srtm.csi.cgiar.org/SELECTION/inputCoord.asp). All the calculations were performed using MatLab and the parameters for the earth are offered by the Mapping Toolbox 3. Because area and species richness did not have a linear relationship, log-transformed area was used as the explanatory variable [46], [47]. Thus, the proportion of total area found at intervals of 100 m in study zone was expressed as the percentage of the total area considered in this study, showing a hump-shaped pattern in the elevation mid-point (peak at the range between 2500 to 2600 m) (Figure S2e).

#### Foods and antagonists

Insect larvae were considered as an important foods, and ants as antagonists for epigaeic beetles [48], [49]. We obtained insect larva and antagonist data from the pitfall trapping as the same trapping regime as epigaeic beetles (Figure S2i, j). However, pitfall trapping is not the best way to measure the potential foods or antagonists of epigaeic beetles, so results from these data were treated with caution.

### Analysis

Polynomial regression analyses were used to ascertain the distribution patterns for the estimates of species richness (rarefied, Chao2 and interpolated) along the elevation gradient. Linear and quadratic models were compared by the Akaike Information Criterion (AIC, lower AIC value means better fit of the model) [50] using statistical package R version 2.15.2 [51].

The relationship between the measures of species richness and the explanatory variables was examined for each individual variable using simple ordinary least squares (OLS) regressions. We then used multiple linear regressions to explore multivariate explanations for testing how well these explanatory variables predicted the species richness patterns. Among the set of factors, we selected five variables, temperature, area, litter cover, insect larva density and ant density. Because precipitation, PET and AET were highly correlated with one another and with temperature, and woody plant species and canopy cover were strongly correlated with litter cover, we dropped these factors from the model and used temperature and litter cover only (Tables S3, S4). Following the Akaike information criterion (AIC) for statistics [50], the best model was selected from a total of 31 OLS models formed with all possible combinations of five individual explanatory variables. All analyses including correlation and regressions were performed using SAM 4.0 [52].

However, such a linear model are relevant only for linear relationships between the potential explanatory variables and diversity, and could not be fit for several plausible scenarios under which a unimodal model is actually more biologically reasonable. Thus, we also examine a unimodal model to detect such curvilinear relationships. To do this, we included a quadratic term into the regression function (*Y* = *b*_0_+*b*_1_ *X*+*b*_2_ *X* ^2^, with *Y* : dependent variable, *X*: independent variable and *b*_n_: coefficients). This analysis was performed using statistical package R version 2.15.2 [51].

Geographical data such as those in our study were generally spatially autocorrelated, and thus can cause non-significant relationships to appear significant when using traditional statistical approaches. To correct for spatial autocorrelation in regression residuals, we assessed the potential effects of spatial autocorrelation in three ways following the method by Sanders et al. (2007) [53]. Firstly, we calculated the modified t-test for each regression between the environmental variables and the dependent estimates of beetle species richness according to Dutilleul’s method [54], [55], factoring out the effects of spatial autocorrelation, and reporting adjusted *P*-values (*P*_adj_ for *r^2^*) based on the effective degrees of freedom in the results. Secondly, we calculated Moran’s *I* across eight spatial distance classes for species richness to test whether any of the response or predictor variables were spatially autocorrelated [55], using SAM 4.0 [52]. Third, to examine whether the residuals from the best models for multiple regressions described above were spatially autocorrelated, we calculated Moran’s *I* on them. If no spatial autocorrelation was found in the residuals of the model including the environmental factors, then there is no statistical bias introduced by spatial autocorrelation in the original regression [55].

### Ethical Considerations

All specimens used in this study were neither endangered nor protected species, and no specific permits were required for the described field studies. Also, the specimens were collected with the permission by Wolong Natural Reserve (see Acknowledgement).

## Results

### Species Richness Pattern along the Elevational Gradient

**Figure 2:**
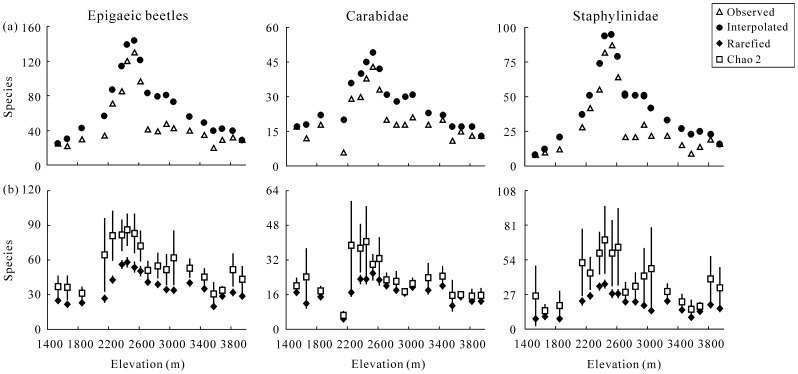
Elevational pattern of richness estimates of epigaecic beetles (including Carabidae and Staphylinidae) along the elevational gradients at east slope of Mt. Balang. (a) Observed and interpolated (empirical) species richness, (b) Rarefied richness and Chao2 estimated richness (with standard error).

A total of 74416 epigaeic beetles belonging to 260 species were sampled within the 58 studied plots at 19 sites along the elevational gradient (Table S1). A full list is provided in Table S2. Of the specimens sampled, 63883 (87 species) were identified to the family Carabidae, and 10533 (173 species) to the family Staphylinidae. The number of species observed at a single site varied from 22 to 130 for whole epigaeic beetles, 6 to 43 for Carabidae, and 8 to 87 for Staphylinidae, exhibiting a mid-elevation peak with the highest number of species observed at approximately 2535 m (Figure 2a).

**Figure 3:**
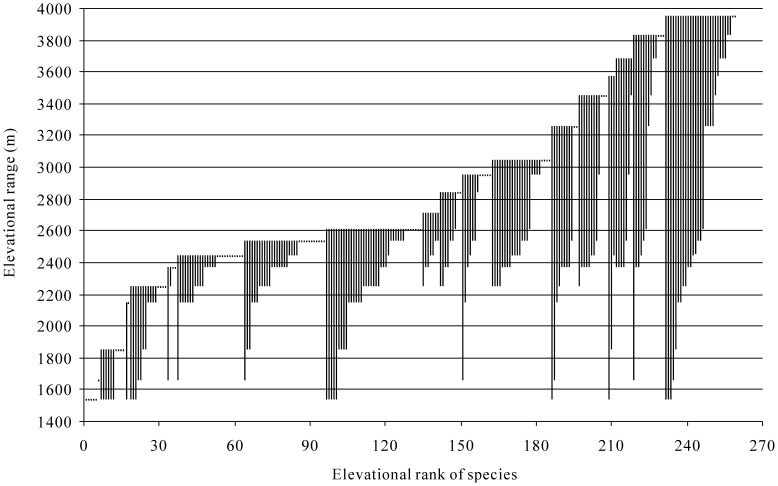
Elevational range sizes of epigaeic beetles at east slope of Mt. Balang. Vertical bars indicate maximum and minimum elevational limits of each species.

**Figure 4:**
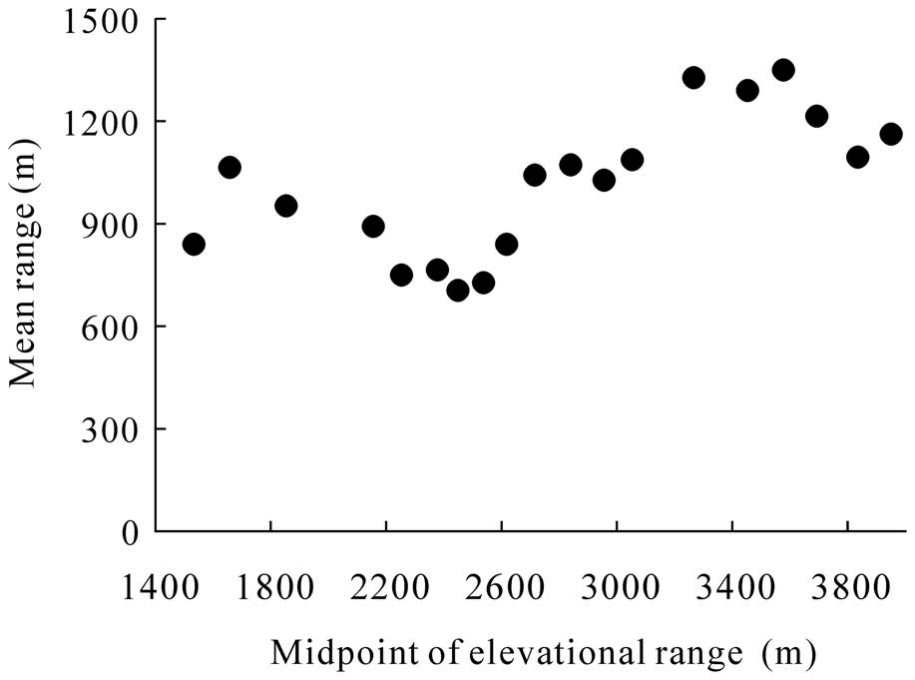
Mean range size of epigaeic beetles along elevational gradient at east slope of Mt. Balang.

Elevational range sizes of the epigaeic beetles showed that most species occupied very narrow elevational ranges along the gradient (Figure 3 and Table S2). Thirty-two beetle species were restricted within 2300 m, whereas 163 species occurred below 3100 m, and 28 species occurred only above 3000 m (Figure 3). Approximately 63% (165 spp.) of the beetle species were detected at elevational ranges of <500 m, but only 7% (18 spp.) at the ranges of >1500 m. In addition, there were 25% (64 spp.) of beetle species found at only a single elevation, but three species (*Pterostichus expedita*, *Pterostichus noguchi* and *Philonthus azuripennis*) occurred at the whole elevational gradient (elevational range = 2415 m) (Table S2). The range sizes of low elevation species (especially those occurring below 2500 m) tended to decrease with elevation (*r* = - 0.738, *P* = 0.058, n = 7), whereas range sizes of high elevation species tended to increase with elevation (*r* = 0.705, *P* = 0.010, n = 12) (Figure 4).

All the estimates of beetle richness which were correlated with one another (*r = *0.838 to 0.923, n = 19, *P*<0.001) showed clear hump-shaped patterns ranging from *c.* 2375 m to 2615 m along the elevational gradient (Figure 2). The family Carabidae and Staphylinidae perfectly reflects the richness pattern of the whole epigaeic beetles (Figure 2).

Polynomial (quadratic) and linear models were compared to test if polynomial regressions may have better explained variation in species richness than did simple linear regressions. Following the rule of the lowest AIC, polynomial regressions fit the data significantly better than did simple linear regressions for all measures of species richness of all taxa (Table 1).

**Table 1:** Polynomial regressions of richness patterns.

	Regressions	Rarefied	Chao 2	Interpolated
Epigaeic beetles	Linear model	0.004	0.064	0.034
	(*r* ^2^, AIC)	5.173	7.924	7.458
	Quadratic model	0.469	0.378	0.645
	(*r* ^2^, AIC)	4.702	7.673	6.616
Carabidae	Linear model	0.006	0.071	0.080
	(*r* ^2^, AIC)	3.502	5.474	4.945
	Quadratic model	0.290	0.276	0.657
	(*r* ^2^, AIC)	3.324	5.383	4.115
Staphylinidae	Linear model	0.002	0.068	0.020
	(*r* ^2^, AIC)	4.427	7.376	6.804
	Quadratic model	0.431	0.332	0.632
	(*r* ^2^, AIC)	4.022	7.201	5.984

Rarefied, Chao 2 and interpolated richness patterns of epigaeic beetles and family Carabidae and Staphylinidae were considered along the elevational gradient at the east slope of Mt. Balang.

### Regression on Environmental Variables

Along the elevational gradient, all three estimates of species richness of the epigaeic beetles as a whole were correlated strongly with the litter cover and area, but only rarefied and interpolated richness showed statistically significant correlation with the two factors and explained more than 47% of variation (Table 2). The family Carabidae and Staphylinidae also showed the similar tendency in species richness responding to environmental variables. Litter cover was significantly correlated for the three estimates of carabid richness and interpolated richness of the staphylinids, but area showed stronger explanatory power in the two patterns of rarefied and interpolated richness of Staphylinidae (Table 2). In addition, the abundance of potential antagonists (ants) was negatively correlated with all estimates of species richness of the three taxa, and showed statistically significant correlation with rarefied richness of carabids only (Table 2). The multivariate results indicated that temperature and area were the most important factors for Chao 2 and interpolated richness of the three taxa (except Chao 2 of carabids), and the area only determined rarefied richness of staphylinids (Table 3). In addition, litter cover was still a determinant variable which affected rarefied richness of the whole beetles and Chao 2 richness of carabids alone, and rarefied richness of carabids together with insect larvae (Table 3).

**Table 2:** Simple OLS regression analysis.

	Rarefied		Chao 2		Interpolated	
	*r* ^2^	*P*_adj_	*r* ^2^	*P*_adj_	*r* ^2^	*P*_adj_
Epigaeic beetles						
Temperature	0.004	0.678	0.038	0.374	0.034	0.520
Log Area	**0.473**	**0.036**	0.340	0.078	**0.594**	**0.043**
Litter cover	**0.521**	**0.030**	0.251	0.140	**0.542**	**0.045**
Insect larvae	0.009	0.749	0.099	0.316	0.095	0.415
Ants	0.247 (−)	0.132	0.133 (−)	0.305	0.256 (−)	0.215
Carabidae						
Temperature	0.006	0.739	0.086	0.296	0.081	0.435
Log Area	0.209	0.064	0.181	0.103	0.514	0.068
Litter cover	**0.500**	**0.004**	**0.287**	**0.038**	**0.517**	**0.049**
Insect larvae	0.002	0.873	0.051	0.402	0.094	0.448
Ants	**0.334 (**−**)**	**0.016**	0.174 (−)	0.139	0.350 (−)	0.158
Staphylinidae						
Temperature	0.002	0.499	0.068	0.282	0.020	0.572
Log Area	**0.506**	**0.042**	0.300	0.108	**0.615**	**0.036**
Litter cover	0.364	0.101	0.196	0.205	**0.540**	**0.044**
Insect larvae	0.039	0.574	0.097	0.328	0.093	0.403
Ants	0.127 (−)	0.312	0.120 (−)	0.342	0.216 (−)	0.243

Rarefied, Chao 2 and interpolated richness were analyzed with five environmental factors for three beetle taxa. *P*_adj_ is the *P*-value for *r* ^2^, based on degrees of freedom adjusted to account for spatial autocorrelation using Dutilleul’s (1993) method. Bold faced entries indicate significant *r* ^2^ (*P*_adj_) <0.05.

**Table 3:** Multiple regressions analysis.

	Rarefied	Chao 2	Interpolated
Epigaeic beetles			
Model fit	0.521	0.471	0.701
(*r* ^2^, AIC)	139.897	198.468	178.216
Temperature (beta)		0.369	0.332
Log area (beta)		0.648	0.830
Litter cover (beta)	0.722		
Carabidae			
Model fit	0.579	0.287	0.688
(*r* ^2^, AIC)	109.012	154.499	132.199
Temperature (beta)			0.424
Log area (beta)			0.792
Litter cover (beta)	0.800	0.536	
Insect larvae	−0.297		
Staphylinidae			
Model fit	0.506	0.433	0.697
(*r* ^2^, AIC)	126.244	189.469	165.744
Temperature (beta)		0.370	0.291
Log area (beta)	0.712	0.614	0.836

Rarefied, Chao 2 and interpolated richness were analyzed with five environmental factors for three beetle taxa. Model selection (*best model*) for multiple regressions (which do not account for spatial autocorrelation) was based on minimizing AIC, with consideration of all possible models. For comparison with the best model, *r* ^2^ and AIC are also shown. *Beta* is the standardized regression slope for each factor in the best model.

Some variables were poorly correlated with species richness in the linear regressions (Table 2), but highly correlated with species richness when applying second order polynomial regressions models (Table 4). This was especially true for temperature, for which regression values increased from 0.002–0.086 (all cases, not significant) in linear models to 0.276 and 0.657 in curvilinear models (most cases, *P*<0.05).

**Table 4:** Second order polynomial regression analysis.

	Rarefied		Chao 2		Interpolated	
	*r* ^2^	*P*_adj_	*r* ^2^	*P*_adj_	*r* ^2^	*P*_adj_
Epigaeic beetles						
Temperature	**0.469**	**0.006**	**0.378**	**0.022**	**0.645**	**<0.001**
Log Area	**0.658**	**<0.001**	**0.537**	**0.002**	**0.806**	**<0.001**
Litter cover	**0.644**	**<0.001**	**0.420**	**0.013**	**0.648**	**<0.001**
Insect larvae	0.024	0.823	0.104	0.417	0.120	0.361
Ants	0.267	0.083	0.143	0.290	0.289	0.065
Carabidae						
Temperature	0.290	0.064	0.276	0.075	**0.657**	**<0.001**
Log Area	**0.435**	**0.010**	**0.409**	**0.015**	**0.777**	**<0.001**
Litter cover	**0.545**	**0.002**	**0.382**	**0.021**	**0.667**	**<0.001**
Insect larvae	0.083	0.496	0.094	0.453	0.170	0.226
Ants	**0.500**	**0.004**	0.176	0.212	**0.377**	**0.023**
Staphylinidae						
Temperature	**0.431**	**0.011**	**0.332**	**0.040**	**0.632**	**<0.001**
Log Area	**0.612**	**<0.001**	**0.473**	**0.006**	**0.803**	**<0.001**
Litter cover	**0.500**	**0.004**	**0.351**	**0.032**	**0.629**	**<0.001**
Insect larvae	0.029	0.792	0.101	0.425	0.105	0.412
Ants	0.130	0.328	0.135	0.315	0.252	0.098

Rarefied, Chao 2 and interpolated richness were analyzed with five environmental factors for three beetle taxa. Bold faced entries indicate significant *r* ^2^ (*P*_adj_) <0.05.

Little evidence of spatial autocorrelation was found in three estimates of species richness patterns, except for interpolated richness of all beetles and carabids at the smallest distance or of carabids at larger distance (the sixth distance unit) classes, where interpolated richness was positively spatially autocorrelated in the smallest distance classes and negatively spatially autocorrelated in the larger distance (Table S5). Fitting the best models of three estimates of species richness patterns including temperature, area, litter cover, foods (insect larvae) and antagonists (ants) removed all of the significant spatial autocorrelation in the richness data across all distance classes (Table S6). This confirms that the environmental variables, especially for temperature, area and litter cover, which together contributed more than 40% of spatial variation in species richness patterns, drive the elevational diversity gradients in epigaeic beetles.

## Discussion

### Richness Patterns

Our study displayed a distinct mid-elevation peak at the elevational range between 2500 to 2600 m (*c.* 2535 m) in species richness pattern of epigaeic beetles at the east slope of Mt. Balang. This result supported the existence of hump-shaped patterns in diversity along elevational transects, which are regarded as a general elevational richness pattern [8], [9], [10] and supported by many studies from other insects over the past 25 years [19], [20], [22], [23], [37], [56]. In addition, other taxa in the Tibetan Plateau and nearby regions were also frequently documented as having unimodal patterns in species richness, e.g. small mammals in Mt. Qilian in China [57], birds in Himalaya in India [58], fishes, amphibians and reptiles in Hengduan Moutains of Tibetan Plateau in China [59], [60], and plants in Mt. Gaolinggong in south-east Tibet of China [61].

Compared with the unimodal patterns in the above insect studies, the relatively low values for species numbers at the two ends of the elevational gradient resulted in a sharper peak in the middle elevation in richness pattern in our study. Human disturbance might be a possible explanation for the sharper peak. In our study, human disturbance occurred usually in the lower elevations (4 samples below 2200 m disturbed by agriculture) or higher elevations (6 samples above 3200 m slightly disturbed by tourism) of the elevational gradient might reduce the species diversity, as our previous studies suggested at more local scales in the study region [62], [63]. However, we note that even if these disturbed sites were extracted, the remaining elevational bands still showed a mid-elevation peak and would not qualitatively change the overall patterns we document here (Figure 2). We feel confident, therefore, that such disturbance or sampling scope is unlikely to have biased the analyses presented here.

### Richness Patterns and Environmental Variables

As Sanders & Rahbek (2012) summarized [8], a number of factors have been proposed to explain elevational distributions of species richness patterns, and some of the most frequently tested included climate, area, geometric constraints, productivity, species pool, disturbance, habitat heterogeneity and evolutionary history [1], [17], [18], [19], [24], [47]. In our study, we tested two alternative groups of explanatory factors (environment vs. direct biotic interactions), and found strong explanatory power of environment factors. In particular, when we examined the potential correlates of species richness in isolation of one another using simple linear or second order polynomial regressions, we found that species richness of beetles were positively and strongly correlated with temperature (climate variable), area (spatial variable) and litter cover (habitat heterogeneity) (Tables 2, 4), all of which were also in combination with other variables in the multiple regressions (Table 3).

Habitat heterogeneity, area and productivity are often correlated with insect species richness at various geographical scales [8], [19], [22], [37], [53], [64], associated with most frequently posited mechanisms such as the ‘more individuals hypothesis’ [65], ‘species-area relationship hypothesis’ [1], metabolic theory of ecology (MTE) [12] or ‘species-energy’ theory [66]. Firstly, as a representative of habitat heterogeneity, a positive relationship between litter cover and the diversity of epigaeic beetles might be a general rule. Our previous studies have found that the coverage and depth of litter layer determined the species richness and abundance of epigaeic beetles in the studied region [62], [63], [67], [68], [69]. Because larger average population sizes (abundance) can reduce the probability of local extinction (“abundance-extinction mechanism”) [65], [70], or as more individuals are supported, the probability of a novel species being ‘sampled’ by a local assemblage from the regional species pool increases (“sampling mechanism”) [71], [72], through changing the abundance of beetles along the elevational gradient, the litter cover might indirectly shape the elevational pattern in species richness of epigaeic beetles. Secondly, area is always a crucial parameter determining biodiversity patterns and can affect species richness both indirectly and directly [2], [14]. Species-area relationships predicted that as area increases, so does species richness [1]. Therefore, larger area in the middle elevations in our study would result in more species in mid-elevations. Thirdly, temperature usually covariates with net primary productivity (NPP) [66], [73], limits the physiology, behaviour or ranges of individuals [70], [74] or drives speciation rates [12], [75], [76], so it is often considered as an important climate factor to indirectly limit diversity. In our study, annual temperature with a monotonic linear decreasing pattern could not exhibit strong correlations with the hump-shaped pattern of species richness of beetles along the elevational gradient in simple linear regressions (Table 2), but showed strong correlations with richness patterns in second order polynomial regressions or in combination with other variables in multiple regressions (Table 4).

Although food availability and abundance of antagonists were two important factors determining the habitat and microhabitat preference of epigaeic beetles [48], [49], compared with the environmental factors, these two direct biological interactions showed only a weak explanatory power of beetle richness in this study, apart from the strongly negative correlations between antagonists and richness of carabids (Table 2). Two possible aspects might result in the weak support. Firstly, the abundance of food was sufficient for beetles and the density of antagonists was low, so the foods and antagonists would not strongly impact the richness of beetles. However, ants as the important antagonists for carabids usually preferred the microhabitat of sun exposure (negatively correlated with litter cover) [77], [78], thus the high density of ants might also reduce the richness and abundance of carabids in low and high elevations. Secondly, the rough data for foods and antagonists based on the pitfall traps designed for capturing beetles were not accurate, so the quality of these data was insufficient in discerning distribution patterns.

### Conclusions

In sum, epigaeic beetles showed a hump-shaped pattern along the elevational gradient at the east slope of Mt. Balang, peaking approximately at the elevational range between 2400 m to 2600 m (*c.* 2535 m), in the Eastern Tibetan Plateau. Environmental factors are likely to account for most variations in the observed richness peaks of epigaeic beetles at mid-elevations, but direct biological interactions only for carabid richness patterns. Marked differences occurred between different taxa, making it impossible to explain all patterns for with a single key factor [37], [47], [56], with temperature, area and litter cover together able predict beetle species richness for nearly all richness patterns. In addition, the small range sizes of species along the whole gradient suggest that conservation efforts should consider the entire gradient rather than just mid-elevations.

## Supporting Information

Figure S1 Species accumulation curves of epigaeic beetles. Curves based on number of individuals detected in different sampling sites along the elevational gradients at east slope of Mt. Balang. Numbers in the figures indicate elevation (m) of the sampling site.
(TIF)

Figure S2 Environmental variables considered for this study. (a) Temperature, (b) Precipitation, (c) Potential evapotranspiration (PET), (d) Actual evapotranspiration (AET), (e) Woody plant species, (f) Canopy cover, (g) Litter cover, (h) Area, (i) Mean density (number of individuals per sample at each elevation site) of insect larvae, and (j) Mean density (number of individuals per sample at each elevation site) of ants.
(TIF)

Table S1 Samples, trapping locations, traps and numbers of species and individuals collected at 19 studied elevations.
(DOC)

Table S2 Midpoint and range of 260 epigaeic beetle species recorded.
(DOC)

Table S3 Reduction of dimension: factor analysis reduced variability within explanatory variables to two dimensions. High factor loadings in the same dimension indicate possible collinearity within the variable groups.
(DOC)

Table S4 Pearson correlation coefficient among ten environmental variables.
(DOC)

Table S5 Tests of spatial autocorrelation on the beetle diversity at three estimates of species richness. Significant values (after Bonferroni adjustment of the critical α to 0.006 to correct for multiple tests) are in bold and indicated with an ‘*’.
(DOC)

Table S6 Tests of spatial autocorrelation on the residuals of the multiple regression model on species richness. Explanatory variables included temperature, litter cover, area, foods (insect larvae) and antagonists (ants). Significant values were set as the critical α to 0.006 to correct for multiple tests.
(DOC)
