# Supplementary material for: Elevational Gradient in Species Richness Pattern of Epigaeic Beetles and Underlying Mechanisms at East Slope of Balang Mountain in Southwestern China
Source: PLoS One. 2013 Jul 18;8(7):e69177. doi: 10.1371/journal.pone.0069177 (PMC3715450; doi:10.1371/journal.pone.0069177)
Supplement: Table S1 — Samples, trapping locations, traps and numbers of species and individuals collected at 19 studied elevations. (DOC) [file pone.0069177.s003.doc]

**Table S1. Samples, trapping locations, traps and numbers of species and individuals collected at 19 studied elevations.**

| Elevation (m) | Samples (plots) | Trapping locations (subplots) | Number of traps | Number of species | Number of sampled individuals |
| --- | --- | --- | --- | --- | --- |
| 1535 | 1 | 5 | 25 | 25 | 341 |
| 1660 | 1 | 5 | 25 | 22 | 196 |
| 1850 | 2 | 10 | 50 | 30 | 53, 482 |
| 2150 | 2 | 8 | 40 | 34 | 85, 133 |
| 2250 | 4 | 16 | 80 | 71 | 116, 324, 154, 414 |
| 2375 | 4 | 16 | 80 | 85 | 202, 1179, 771, 168 |
| 2445 | 9 | 36 | 180 | 120 | 826, 432, 196, 196, 619, 496, 216, 1313, 741 |
| 2535 | 15 | 67 | 335 | 130 | 505, 735, 882, 545, 422, 2869, 1378, 1970, 2369, 1681, 1522, 1186, 1081, 5427, 529 |
| 2615 | 7 | 35 | 175 | 97 | 2839, 2384, 643, 375, 575, 2833, 416 |
| 2710 | 1 | 5 | 25 | 41 | 3045 |
| 2840 | 1 | 5 | 25 | 39 | 3253 |
| 2955 | 3 | 15 | 75 | 48 | 2730, 1552, 2200 |
| 3050 | 2 | 10 | 50 | 43 | 4203, 3596 |
| 3260 | 1 | 5 | 25 | 40 | 230 |
| 3450 | 1 | 5 | 25 | 35 | 1357 |
| 3570 | 1 | 5 | 25 | 20 | 3086 |
| 3685 | 1 | 5 | 25 | 29 | 2445 |
| 3830 | 1 | 5 | 25 | 32 | 3329 |
| 3950 | 1 | 5 | 25 | 29 | 571 |
| Total | 58 | 263 | 1315 | 260 | 74416 |
