# Supplementary material for: Elevational Gradient in Species Richness Pattern of Epigaeic Beetles and Underlying Mechanisms at East Slope of Balang Mountain in Southwestern China
Source: PLoS One. 2013 Jul 18;8(7):e69177. doi: 10.1371/journal.pone.0069177 (PMC3715450; doi:10.1371/journal.pone.0069177)
Supplement: Table S2 — Midpoint and range of 260 epigaeic beetle species recorded. (DOC) [file pone.0069177.s004.doc]

**Table S2. Midpoint and range of 260 epigaeic beetle species recorded.**

| Species name | Individuals | Midpoint (m) | Range (m) |
| --- | --- | --- | --- |
| **Carabidae** |  |  |  |
| **Broscinae** |  |  |  |
| *Broscodera dreuxi* Deuve | 2 | 3050 | 0 |
| **Carabinae** |  |  |  |
| *Calosoma davidis* Gehin | 16 | 2050 | 400 |
| *Carabus (Apotomopterus) ascendens* Semenow | 19 | 1657.5 | 315 |
| *Carabus (Archaeocarabus) shamaevi* Imura | 861 | 2232.5 | 765 |
| *Carabus (Aristocarabus) viridifossulatus* Fairmaire | 19 | 2992.5 | 915 |
| *Carabus (Neoplesius) sichuanicola* Deuve | 717 | 2747.5 | 605 |
| *Carabus (Neoplesius)* sp. | 543 | 3640 | 380 |
| *Cychropsis draconis* Deuve | 81 | 2850 | 1200 |
| *Cychropsis korelli* Kleinfeld | 35 | 3182.5 | 1295 |
| *Cychrus okamotoi* Imura, Su & Osawa | 680 | 2912.5 | 1075 |
| *Cychrus* sp. | 11 | 2455 | 160 |
| **Harpalinae** |  |  |  |
| *Agonum* sp.1 | 2 | 2250 | 0 |
| *Agonum* sp.2 | 39 | 2650 | 800 |
| *Agonum* sp.3 | 5 | 2347.5 | 195 |
| *Agonum* sp.4 | 2 | 2455 | 160 |
| *Agonum* sp.5 | 1 | 1850 | 0 |
| *Agonum* sp.6 | 335 | 2542.5 | 335 |
| *Amara macronota* (Solsky) | 7 | 2397.5 | 1725 |
| *Amara* sp.1 | 172 | 2075 | 1080 |
| *Amara* sp.2 | 4 | 3260 | 0 |
| *Amara* sp.3 | 25 | 2075 | 1080 |
| *Amara* sp.4 | 1 | 2445 | 0 |
| *Amara* sp.5 | 6 | 3757.5 | 145 |
| *Andrewesius coeruleatus* Farimaire | 7773 | 3242.5 | 1415 |
| *Andrewesius glasaour* Morvan | 203 | 2947.5 | 1005 |
| *Anisodactylus punctatipennis* Morawitz | 44 | 1892.5 | 715 |
| *Aristochroa panda* Tian | 5853 | 2817.5 | 885 |
| *Chlaenius micans* (Fabricius) | 107 | 1535 | 0 |
| *Chydaeus bedeli* (Tschitscherine) | 8 | 2347.5 | 195 |
| *Dolichus halensis* Schaller | 242 | 1892.5 | 715 |
| *Dromius* sp. | 2 | 2312.5 | 125 |
| *Harpalus fokienensis* Schauberger | 8 | 1692.5 | 315 |
| *Harpalus pastor* Motschulsky | 1 | 1535 | 0 |
| *Harpalus tridens* Morawitz | 267 | 1892.5 | 715 |
| *Harpalus* sp.1 | 1 | 2250 | 0 |
| *Harpalus* sp.2 | 9 | 1535 | 0 |
| *Harpalus* sp.3 | 12 | 1692.5 | 315 |
| *Lebia* sp. | 1 | 1660 | 0 |
| *Lesticus magnus* (Motschulsky) | 11 | 1692.5 | 315 |
| *Morphodactyla alticola* Bates | 381 | 3100 | 1700 |
| *Myas cavazzutii* Casale & Sciaky | 318 | 2432.5 | 365 |
| *Myas robustus* Fairmaire | 79 | 3197.5 | 1505 |
| *Pristosia* sp.1 | 3 | 2575 | 80 |
| *Pristosia* sp.2 | 4 | 2347.5 | 195 |
| *Pristosia* sp.3 | 5 | 2535 | 0 |
| *Pterostichus aeneocupreus* Fairmaire | 20 | 3202.5 | 1495 |
| *Pterostichus beneshi* Sciaky | 203 | 2650 | 800 |
| *Pterostichus curtatus* Fairmaire | 445 | 2455 | 160 |
| *Pterostichus expedita* Tschitscherine | 4021 | 2742.5 | 2415 |
| *Pterostichus filium* Tschitscherine | 139 | 2432.5 | 365 |
| *Pterostichus haesitatus* Fairmaire | 1785 | 2712.5 | 675 |
| *Pterostichus lanista* Tschitscherine | 185 | 3007.5 | 1125 |
| *Pterostichus montigena* Tschitscherine | 59 | 3002.5 | 95 |
| *Pterostichus noguchi* Bates | 20 | 2742.5 | 2415 |
| *Pterostichus perlutus* Jedicka | 753 | 2382.5 | 465 |
| *Pterostichus pratti* Bates | 17 | 2200 | 100 |
| *Pterostichus straneellus* Jedlicka | 17 | 3002.5 | 95 |
| *Pterostichus* sp.1 | 310 | 2650 | 800 |
| *Pterostichus* sp.2 | 20 | 2552.5 | 2035 |
| *Pterostichus* sp.3 | 3 | 2455 | 160 |
| *Stenolophus* sp. | 5 | 2050 | 400 |
| *Stomis vignai* Sciaky | 313 | 2432.5 | 365 |
| *Stomis* sp. | 2 | 2410 | 70 |
| *Straneostichus ovipennis* Sciaky | 11 | 2622.5 | 175 |
| *Straneostichus vignai* Sciaky | 25 | 3202.5 | 495 |
| *Synuchus arcuticollis* Motschulsky | 6 | 2075 | 1080 |
| *Synuchus nitidus recticulatus* Lindroth | 192 | 2382.5 | 465 |
| *Synuchus* sp.1 | 1066 | 2542.5 | 335 |
| *Synuchus* sp.2 | 9 | 2392.5 | 285 |
| *Synuchus* sp.3 | 13172 | 3162.5 | 1575 |
| *Trichotichnus* sp.1 | 49 | 2052.5 | 785 |
| *Trichotichnus* sp.2 | 81 | 2432.5 | 365 |
| *Trichotichnus* sp.3 | 6 | 1955 | 590 |
| *Trichotichnus* sp.4 | 74 | 2900 | 2100 |
| *Xestagonum nitouensis* (Jedlicka) | 10705 | 2805 | 2290 |
| *Xestagonum wassulandi* (Jedlicka) | 9389 | 3030 | 1310 |
| **Loricerinae** |  |  |  |
| *Loricera mirabilis* Jedlicka | 2 | 3450 | 0 |
| **Nebriinae** |  |  |  |
| *Leistus wolong* Farkac & Sciaky | 94 | 3242.5 | 1415 |
| *Leistus* sp.1 | 1693 | 2712.5 | 675 |
| *Leistus* sp.2 | 20 | 3700 | 500 |
| *Nebria* sp. | 1 | 3830 | 0 |
| *Notiophilus schawalleri* Barsevskis | 38 | 3760 | 380 |
| **Trechinae** |  |  |  |
| *Bembidion* sp. | 5 | 3757.5 | 145 |
| *Patrobus* sp. | 5 | 2535 | 0 |
| *Trechus* sp.1 | 2 | 2897.5 | 725 |
| *Trechus* sp.2 | 5 | 2687.5 | 305 |
| *Trechus* sp.3 | 1 | 3950 | 0 |
| **Staphylinidae** |  |  |  |
| **Apateticinae** |  |  |  |
| *Apatetica* sp. | 1 | 2445 | 0 |
| **Micropeplinae** |  |  |  |
| *Micropeplus dentatus* Zhao et Zhou | 6 | 2745 | 420 |
| **Omaliinae** |  |  |  |
| *Acrolocha* sp. | 9 | 2622.5 | 175 |
| Anthophagini, sp.1 | 1 | 2535 | 0 |
| Anthophagini, sp.2 | 1 | 2840 | 0 |
| *Eudectus* sp.1 | 19 | 2745 | 420 |
| *Eudectus* sp.2 | 1 | 2615 | 0 |
| Eusphalerini, sp. | 1 | 3050 | 0 |
| *Eusphalerum* sp.1 | 4 | 2490 | 90 |
| *Eusphalerum* sp.2 | 5 | 2747.5 | 605 |
| *Omalium rivulare* (Paykull) | 19 | 2700 | 510 |
| *Unamis* sp. | 4 | 2575 | 80 |
| **Osoriinae** |  |  |  |
| *Osorius hauseri* Bernhauer | 49 | 2232.5 | 765 |
| **Oxytelinae** |  |  |  |
| *Anotylus hirtulus* (Eppelsheim) | 11 | 1692.5 | 315 |
| *Anotylus nitidifrons* (Wollaston) | 73 | 2392.5 | 285 |
| *Anotylus sculpturatus* Gravenhorst | 821 | 2712.5 | 675 |
| *Anotylus* *tetracarinatus* (Block) | 180 | 2075 | 1080 |
| *Anotylus* sp.1 | 2 | 2410 | 70 |
| *Anotylus* sp.2 | 1 | 2535 | 0 |
| *Anotylus* sp.3 | 117 | 2480 | 460 |
| *Anotylus* sp.4 | 1 | 2615 | 0 |
| *Anotylus* sp.5 | 1 | 2445 | 0 |
| *Anotylus* sp.6 | 3 | 2232.5 | 765 |
| *Anotylus* sp.7 | 1 | 2535 | 0 |
| *Oxytelus dohertyi* Cameron | 28 | 2747.5 | 605 |
| *Oxytelus* *robustus* Schubert | 3 | 3890 | 120 |
| *Oxytelus* *ailaoshanicus* Lü & Zhou | 31 | 2432.5 | 365 |
| *Platystethus* *erlangshanus* Yan, Li & Zheng | 6 | 2495 | 240 |
| **Paederinae** |  |  |  |
| *Paederus chinensis* Berhauor | 278 | 2460 | 1600 |
| *Stilicoderus* sp. | 73 | 2817.5 | 885 |
| **Phloeocharinae** |  |  |  |
| *Charhyphus* sp. | 2 | 2490 | 90 |
| **Piestinae** |  |  |  |
| *Siagonium* sp. | 1 | 2615 | 0 |
| **Phloeocharinae** |  |  |  |
| *Megarthrus* sp.1 | 6 | 2445 | 0 |
| *Megarthrus* sp.2 | 8 | 2650 | 800 |
| *Megarthrus* sp.3 | 1 | 3950 | 0 |
| **Pselaphinae** |  |  |  |
| *Saltysedes* sp. | 108 | 2382.5 | 465 |
| Batrisini, sp.1 | 64 | 2382.5 | 465 |
| Batrisini, sp.2 | 9 | 23825 | 465 |
| Batrisini, sp.3 | 9 | 2665 | 580 |
| Tyrini, sp. | 15 | 2342.5 | 385 |
| **Pseudopsinae** |  |  |  |
| *Pseudopsis* sp. | 8 | 2455 | 160 |
| **Scaphidiinae** |  |  |  |
| *Scaphidium vicinum* Pic | 2 | 2410 | 70 |
| *Scaphidium* sp. | 25 | 2297.5 | 295 |
| **Staphylininae** |  |  |  |
| *Craspedomerus beckeri* Bernhauer | 8 | 2075 | 1080 |
| *Craspedomerus gigante* Li & Zhou | 256 | 2552.5 | 805 |
| *Dinothenarus balangensis* Yang and Zhou | 22 | 3817.5 | 265 |
| *Dinothenarus sagaris* Smetana | 32 | 3137.5 | 1385 |
| *Eucibdelus* sp.1 | 1 | 2445 | 0 |
| *Eucibdelus* sp.2 | 1 | 2445 | 0 |
| *Eucibdelus* sp.3 | 21 | 3030 | 1310 |
| *Gabrius fimetarioides* (Scheerpeltz) | 2 | 2490 | 90 |
| *Gabronthus* sp. | 1 | 3260 | 0 |
| *Indoquedius aculeus* Zhao et Zhou | 7 | 2200 | 100 |
| *Indoquedius* sp. | 2 | 2392.5 | 285 |
| *Miobdelus* sp. | 14 | 3030 | 1310 |
| *Ocypus* sp.1 | 2509 | 2545 | 590 |
| *Ocypus* sp.2 | 219 | 3605 | 690 |
| *Ocypus* sp.3 | 6 | 1850 | 0 |
| *Ocypus* sp.4 | 961 | 3102.5 | 1455 |
| *Ocypus* sp.5 | 140 | 3282.5 | 1335 |
| *Ocypus* sp.6 | 566 | 3050 | 1800 |
| *Ocypus* sp.7 | 2 | 2575 | 80 |
| *Ocypus* sp.8 | 9 | 2792.5 | 515 |
| *Ocypus* sp.9 | 7 | 2392.5 | 285 |
| *Ocypus* sp.10 | 3 | 2577.5 | 265 |
| *Ocypus* sp.11 | 1 | 1535 | 0 |
| *Othius* sp.1 | 80 | 2432.5 | 365 |
| *Othius* sp.2 | 4 | 2392.5 | 285 |
| *Othius* sp.3 | 3 | 2535 | 0 |
| *Othius* sp.4 | 2 | 2410 | 70 |
| *Philonthus azuripennis* Cameron | 90 | 2742.5 | 2415 |
| *Philonthus coelestis* Bernhauer | 3 | 3817.5 | 265 |
| *Philonthus emdeni* Bernhauer | 1 | 2615 | 0 |
| *Philonthus lan* Schillhammer | 1 | 2535 | 0 |
| *Philonthus mercurii* Tikhomirova | 1 | 2250 | 0 |
| *Philonthus purpuripennis* Reitter | 5 | 3817.5 | 265 |
| *Philonthus saphyreus* Schillhammer | 181 | 3050 | 1800 |
| *Philonthus* sp.1 | 2 | 2200 | 100 |
| *Philonthus* sp.2 | 1 | 3830 | 0 |
| *Philonthus* sp.3 | 2 | 2200 | 100 |
| *Philonthus* sp.4 | 4 | 2745 | 2170 |
| *Philonthus* sp.5 | 4 | 3545 | 570 |
| *Philonthus* sp.6 | 10 | 3890 | 120 |
| *Philonthus* sp.7 | 10 | 2297.5 | 295 |
| *Philonthus* sp.8 | 8 | 3605 | 690 |
| *Philonthus* sp.9 | 1 | 2250 | 0 |
| *Philonthus* sp.10 | 1 | 3830 | 0 |
| *Philonthus* sp.11 | 8 | 2297.5 | 295 |
| *Philonthus* sp.12 | 5 | 2817.5 | 885 |
| *Philonthus* sp.13 | 5 | 2297.5 | 295 |
| *Philonthus* sp.14 | 1 | 2445 | 0 |
| *Philonthus* sp.15 | 8 | 2297.5 | 295 |
| *Platydracus* sp.1 | 1 | 2445 | 0 |
| *Platydracus* sp.2 | 3 | 2615 | 0 |
| *Platydracus* sp.3 | 29 | 1692.5 | 315 |
| *Platydracus* sp.4 | 197 | 2232.5 | 765 |
| *Platydracus* sp.5 | 1 | 2615 | 0 |
| *Pseudohesperus tripartus* Li & Zhou | 4 | 2347.5 | 195 |
| *Quedius calvus* Zhao et Zhou | 6 | 2495 | 240 |
| *Quedius capillus* Zhao et Zhou | 5 | 2792.5 | 515 |
| *Quedius dispar* Zhao et Zhou | 1 | 2445 | 0 |
| *Quedius erythras* Smetana | 1 | 2955 | 0 |
| *Quedius inquietus* (Champion) | 1 | 2445 | 0 |
| *Quedius lih* Smetana | 1 | 2840 | 0 |
| *Quedius myau* Smetena | 2 | 3002.5 | 95 |
| *Quedius perlucidus* Zhao et Zhou | 24 | 2577.5 | 265 |
| *Quedius postangulus* Zhao et Zhou | 3 | 2955 | 0 |
| *Quedius propinquus* Zhao et Zhou | 7 | 3320 | 730 |
| *Quedius songpan* Smetana | 11 | 2897.5 | 115 |
| *Quedius supervacuus* Zhao et Zhou | 2 | 3002.5 | 95 |
| *Quedius triumphus* Zhao et Zhou | 1 | 2955 | 0 |
| *Quedius* sp.1 | 40 | 2792.5 | 515 |
| *Quedius* sp.2 | 9 | 2607.5 | 465 |
| *Quedius* sp.3 | 4 | 2832.5 | 435 |
| *Quedius* sp.4 | 7 | 2455 | 160 |
| *Quedius* sp.5 | 1 | 2375 | 0 |
| *Quedius* sp.6 | 5 | 2710 | 1720 |
| *Quedius* sp.7 | 4 | 1850 | 0 |
| *Quedius* sp.8 | 1 | 3050 | 0 |
| *Quedius* sp.9 | 3 | 2955 | 0 |
| *Quedius* sp.10 | 5 | 3605 | 690 |
| *Rientis* sp. | 26 | 3222.5 | 1215 |
| **Steninae** |  |  |  |
| *Stenus* sp.1 | 5 | 1842.5 | 615 |
| *Stenus* sp.2 | 4 | 3567.5 | 235 |
| *Stenus* sp.3 | 4 | 2955 | 0 |
| **Tachyporinae** |  |  |  |
| *Ischnosoma* sp.1 | 7 | 2192.5 | 685 |
| *Ischnosoma* sp.2 | 6 | 2455 | 160 |
| *Lordithon* sp.1 | 4 | 2817.5 | 885 |
| *Lordithon* sp.2 | 8 | 2545 | 590 |
| *Lordithon* sp.3 | 5 | 2575 | 80 |
| *Lordithon* sp.4 | 1 | 2535 | 0 |
| *Lordithon* sp.5 | 1 | 1850 | 0 |
| *Lordithon* sp.6 | 1 | 1535 | 0 |
| *Parabolitobius* sp.1 | 18 | 2747.5 | 605 |
| *Parabolitobius* sp.2 | 1 | 2375 | 0 |
| *Parabolitobius* sp.3 | 1 | 3050 | 0 |
| *Parabolitobius* sp.4 | 2 | 2575 | 80 |
| *Parabolitobius* sp.5 | 2 | 2192.5 | 685 |
| *Sepedophilus* sp.1 | 26 | 2097.5 | 875 |
| *Sepedophilus* sp.2 | 5 | 2017.5 | 715 |
| *Sepedophilus* sp.3 | 3 | 1955 | 590 |
| *Sepedophilus* sp.4 | 7 | 2297.5 | 295 |
| *Sepedophilus* sp.5 | 16 | 2382.5 | 465 |
| *Sepedophilus* sp.6 | 2 | 2535 | 0 |
| *Sepedophilus* sp.7 | 1 | 2445 | 0 |
| *Sepedophilus* sp.8 | 2 | 3110 | 1150 |
| *Sepedophilus* sp.9 | 20 | 2342.5 | 385 |
| *Sepedophilus* sp.10 | 5 | 2410 | 70 |
| *Tachinus* sp.1 | 628 | 2705 | 1110 |
| *Tachinus* sp.2 | 57 | 2432.5 | 365 |
| *Tachinus* sp.3 | 793 | 3030 | 1310 |
| *Tachinus* sp.4 | 415 | 2912.5 | 1075 |
| *Tachinus* sp.5 | 134 | 2755 | 1010 |
| *Tachinus* sp.6 | 16 | 2912.5 | 1075 |
| *Tachinus* sp.7 | 32 | 3605 | 690 |
| *Tachinus* sp.8 | 13 | 2712.5 | 675 |
| *Tachinus* sp.9 | 7 | 2607.5 | 465 |
| *Tachinus* sp.10 | 114 | 2307.5 | 1295 |
| *Tachinus* sp.11 | 16 | 3162.5 | 1575 |
| *Tachinus* sp.12 | 14 | 3102.5 | 1455 |
| *Tachinus* sp.13 | 46 | 2495 | 240 |
| *Tachinus* sp.14 | 126 | 3100 | 1700 |
| *Tachinus* sp.15 | 3 | 2687.5 | 305 |
| *Tachinus* sp.16 | 3 | 2727.5 | 225 |
| *Tachinus* sp.17 | 2 | 2490 | 90 |
| *Tachinus* sp.18 | 1 | 2615 | 0 |
| *Tachinus* sp.19 | 1 | 2535 | 0 |
| *Tachinus* sp.20 | 2 | 2530 | 170 |
| *Tachinus* sp.21 | 1 | 2150 | 0 |
| *Tachyporus* sp.1 | 299 | 2912.5 | 1075 |
| *Tachyporus* sp.2 | 8 | 2947.5 | 1005 |
| *Tachyporus* sp.3 | 3 | 2535 | 0 |
| *Tachyporus* sp.4 | 4 | 2575 | 80 |
| *Tachyporus* sp.5 | 1 | 2535 | 0 |
