# Supplementary material for: Elevational Gradient in Species Richness Pattern of Epigaeic Beetles and Underlying Mechanisms at East Slope of Balang Mountain in Southwestern China
Source: PLoS One. 2013 Jul 18;8(7):e69177. doi: 10.1371/journal.pone.0069177 (PMC3715450; doi:10.1371/journal.pone.0069177)
Supplement: Table S3 — Reduction of dimension: factor analysis reduced variability within explanatory variables to two dimensions. High factor loadings in the same dimension indicate possible collinearity within the variable groups. (DOC) [file pone.0069177.s005.doc]

**Table S3. Reduction of dimension: factor analysis reduced variability within explanatory variables to two dimensions. High factor loadings in the same dimension indicate possible collinearity within the variable groups.**

|  | Dimension 1 | Dimension 2 |
| --- | --- | --- |
| Temperature | -0.879 | -0.465 |
| Precipitation | 0.879 | 0.465 |
| PET | -0.834 | -0.539 |
| AET | -0.929 | -0.344 |
| Area | -0.240 | 0.857 |
| Woody plant species | -0.537 | 0.740 |
| Canopy cover | -0.473 | 0.817 |
| Litter cover | -0.506 | 0.791 |
| Insect larvae | -0.687 | -0.027 |
| Ants | 0.783 | -0.274 |
