# Supplementary material for: Elevational Gradient in Species Richness Pattern of Epigaeic Beetles and Underlying Mechanisms at East Slope of Balang Mountain in Southwestern China
Source: PLoS One. 2013 Jul 18;8(7):e69177. doi: 10.1371/journal.pone.0069177 (PMC3715450; doi:10.1371/journal.pone.0069177)
Supplement: Table S4 — Pearson correlation coefficient among ten environmental variables. (DOC) [file pone.0069177.s006.doc]

**Table S4. Pearson correlation coefficient among ten environmental variables.**

|  | Precipitation | PET | AET | Area | Woody plant species | Canopy cover | Litter cover | Insect larvae | Ants |
| --- | --- | --- | --- | --- | --- | --- | --- | --- | --- |
| Temperature | -1.000 | 0.995 | 0.989 | -0.178 | 0.140 | 0.054 | 0.068 | 0.557 | -0.541 |
| Precipitation |  | -0.995 | -0.989 | 0.178 | -0.140 | -0.054 | -0.068 | -0.557 | 0.541 |
| PET |  |  | 0.971 | -0.266 | 0.071 | -0.026 | -0.004 | 0.529 | -0.476 |
| AET |  |  |  | -0.033 | 0.254 | 0.171 | 0.174 | 0.599 | -0.600 |
| Area |  |  |  |  | 0.734 | 0.780 | 0.696 | 0.223 | -0.330 |
| Woody plant species |  |  |  |  |  | 0.836 | 0.821 | 0.296 | -0.538 |
| Canopy cover |  |  |  |  |  |  | 0.878 | 0.213 | -0.562 |
| Litter cover |  |  |  |  |  |  |  | 0.316 | -0.655 |
| Insect larvae |  |  |  |  |  |  |  |  | -0.393 |
