# Supplementary material for: Elevational Gradient in Species Richness Pattern of Epigaeic Beetles and Underlying Mechanisms at East Slope of Balang Mountain in Southwestern China
Source: PLoS One. 2013 Jul 18;8(7):e69177. doi: 10.1371/journal.pone.0069177 (PMC3715450; doi:10.1371/journal.pone.0069177)
Supplement: Table S5 — Tests of spatial autocorrelation on the beetle diversity at three estimates of species richness. Significant values (after Bonferroni adjustment of the critical α to 0.006 to correct for multiple tests) are in bold and indicated with an ‘*’. (DOC) [file pone.0069177.s007.doc]

**Table S5. Tests of spatial autocorrelation on the beetle diversity at three estimates of species richness.** Significant values (after Bonferroni adjustment of the critical α to 0.006 to correct for multiple tests) are in bold and indicated with an ‘*’.

|  |  |  |  |  | Distance | class |  |  |  |
| --- | --- | --- | --- | --- | --- | --- | --- | --- | --- |
|  |  | 1 | 2 | 3 | 4 | 5 | 6 | 7 | 8 |
| Epigaeic beetles | | | | | | | | | |
| Rarefied | Moran's *I* | 0.533 | -0.161 | -0.118 | -0.345 | 0.089 | -0.613 | 0.159 | <0.001 |
|  | Probability | 0.010 | 0.391 | 0.469 | 0.056 | 0.587 | 0.008 | 0.375 | 0.998 |
| Chao 2 | Moran's *I* | 0.280 | 0.092 | 0.013 | -0.238 | -0.221 | -0.466 | 0.183 | -0.107 |
|  | Probability | 0.130 | 0.615 | 0.934 | 0.184 | 0.218 | 0.024 | 0.343 | 0.337 |
| Interpolated | Moran's *I* | 0.686 | 0.139 | 0.068 | -0.571 | -0.289 | -0.638 | 0.068 | 0.067 |
|  | Probability | **0.006*** | 0.443 | 0.645 | 0.022 | 0.148 | 0.010 | 0.667 | 0.495 |
| Carabidae |  |  |  |  |  |  |  |  |  |
| Rarefied | Moran's *I* | 0.514 | -0.585 | -0.022 | -0.083 | 0.317 | -0.317 | -0.200 | -0.024 |
|  | Probability | 0.008 | 0.014 | 0.862 | 0.533 | 0.110 | 0.118 | 0.236 | 0.778 |
| Chao 2 | Moran's *I* | 0.106 | -0.220 | 0.071 | -0.060 | 0.093 | -0.354 | -0.121 | -0.054 |
|  | Probability | 0.583 | 0.240 | 0.627 | 0.661 | 0.573 | 0.082 | 0.485 | 0.467 |
| Interpolated | Moran's *I* | 0.661 | 0.147 | 0.184 | -0.440 | -0.272 | -0.776 | -0.061 | 0.055 |
|  | Probability | **0.004*** | 0.413 | 0.251 | 0.028 | 0.144 | **0.002*** | 0.701 | 0.561 |
| Staphylinidae |  |  |  |  |  |  |  |  |  |
| Rarefied | Moran's *I* | 0.418 | 0.091 | -0.189 | -0.514 | -0.111 | -0.553 | 0.389 | -0.019 |
|  | Probability | 0.018 | 0.551 | 0.275 | 0.024 | 0.493 | 0.010 | 0.064 | 0.846 |
| Chao 2 | Moran's *I* | 0.273 | 0.137 | 0.011 | -0.211 | -0.275 | -0.436 | 0.220 | -0.126 |
|  | Probability | 0.136 | 0.451 | 0.940 | 0.198 | 0.142 | 0.026 | 0.238 | 0.257 |
| Interpolated | Moran's *I* | 0.679 | 0.134 | 0.026 | -0.612 | -0.289 | -0.569 | 0.112 | 0.066 |
|  | Probability | 0.008 | 0.429 | 0.856 | 0.014 | 0.128 | 0.012 | 0.507 | 0.511 |
