# Supplementary material for: Elevational Gradient in Species Richness Pattern of Epigaeic Beetles and Underlying Mechanisms at East Slope of Balang Mountain in Southwestern China
Source: PLoS One. 2013 Jul 18;8(7):e69177. doi: 10.1371/journal.pone.0069177 (PMC3715450; doi:10.1371/journal.pone.0069177)
Supplement: Table S6 — Tests of spatial autocorrelation on the residuals of the multiple regression model on species richness. Explanatory variables included temperature, litter cover, area, foods (insect larvae) and antagonists (ants). Significant values were set as the critical α to 0.006 to correct for multiple tests. (DOC) [file pone.0069177.s008.doc]

**Table S6. Tests of spatial autocorrelation on the residuals of the multiple regression model on species richness.** Explanatory variables included temperature, litter cover, area, foods (insect larvae) and antagonists (ants). Significant values were set as the critical α to 0.006 to correct for multiple tests.

|  |  |  |  |  | Distance | class |  |  |  |
| --- | --- | --- | --- | --- | --- | --- | --- | --- | --- |
|  |  | 1 | 2 | 3 | 4 | 5 | 6 | 7 | 8 |
| Epigaeic beetles | | | | | | | | | |
| Rarefied | Moran's *I* | 0.267 | -0.381 | -0.030 | 0.129 | -0.177 | -0.479 | 0.078 | 0.145 |
|  | Probability | 0.160 | 0.062 | 0.862 | 0.369 | 0.319 | 0.026 | 0.633 | 0.170 |
| Chao 2 | Moran's *I* | 0.242 | -0.185 | 0.060 | 0.033 | <0.001 | -0.483 | 0.010 | -0.019 |
|  | Probability | 0.232 | 0.323 | 0.687 | 0.788 | 1.000 | 0.034 | 0.962 | 0.844 |
| Interpolated | Moran's *I* | 0.170 | -0.261 | 0.028 | 0.117 | -0.134 | -0.480 | 0.054 | 0.081 |
|  | Probability | 0.413 | 0.182 | 0.870 | 0.413 | 0.437 | 0.034 | 0.752 | 0.433 |
| Carabidae |  |  |  |  |  |  |  |  |  |
| Rarefied | Moran's *I* | -0.155 | -0.196 | -0.042 | 0.225 | -0.338 | -0.089 | 0.034 | 0.024 |
|  | Probability | 0.455 | 0.248 | 0.770 | 0.138 | 0.080 | 0.589 | 0.842 | 0.796 |
| Chao 2 | Moran's *I* | 0.048 | -0.046 | 0.162 | -0.070 | -0.320 | -0.376 | 0.008 | 0.038 |
|  | Probability | 0.814 | 0.794 | 0.337 | 0.613 | 0.106 | 0.060 | 0.964 | 0.649 |
| Interpolated | Moran's *I* | 0.092 | -0.249 | 0.022 | 0.100 | -0.123 | -0.390 | -0.032 | 0.094 |
|  | Probability | 0.651 | 0.214 | 0.892 | 0.495 | 0.463 | 0.062 | 0.832 | 0.387 |
| Staphylinidae |  |  |  |  |  |  |  |  |  |
| Rarefied | Moran's *I* | 0.345 | -0.447 | -0.114 | 0.111 | -0.059 | -0.478 | 0.126 | 0.088 |
|  | Probability | 0.078 | 0.04 | 0.479 | 0.405 | 0.743 | 0.022 | 0.439 | 0.395 |
| Chao 2 | Moran's *I* | 0.234 | -0.162 | -0.083 | 0.136 | 0.069 | -0.501 | 0.113 | -0.054 |
|  | Probability | 0.242 | 0.371 | 0.595 | 0.387 | 0.673 | 0.014 | 0.493 | 0.601 |
| Interpolated | Moran's *I* | 0.192 | -0.259 | 0.027 | 0.121 | -0.131 | -0.506 | 0.092 | 0.072 |
|  | Probability | 0.327 | 0.158 | 0.846 | 0.423 | 0.471 | 0.018 | 0.545 | 0.489 |
